# Isolation and detection of microRNA from the egg of chickens

**DOI:** 10.1186/s13104-016-2084-5

**Published:** 2016-05-23

**Authors:** Ben Wade, Michelle Cummins, Anthony Keyburn, Tamsyn M. Crowley

**Affiliations:** School of Medicine, MMR, Bioinformatics Core Research Facility, Deakin University, Pigdons Road, Waurn Ponds, VIC 3216 Australia; CSIRO, Australian Animal Health Laboratory, 5 Portarlington Road, Geelong, VIC 3220 Australia; Poultry Cooperative Research Centre, University of New England, Armidale, NSW 2351 Australia

**Keywords:** microRNA, Chicken, Egg, Yolk, Albumen, Extraction

## Abstract

**Background:**

The egg is a vital part of the chicken developmental process and an important protein source for humans. Despite the chicken egg being a subject of intense research little attention has been given to the role of microRNAs within the egg.

**Findings:**

We report a method for the reproducible and reliable isolation of miRNA from the albumen and yolk of chicken eggs. We also report the detection via real-time PCR of a number of miRNAs from both of these biological fluids.

**Conclusions:**

These findings provide an interesting look into the chicken egg and raise questions as to the role that miRNAs maybe playing in the chicken egg. This method of detecting miRNAs in chicken eggs will allow researchers to investigate the presence of an additional level of epigenetic programming in chick development previously unknown and also how this impacts the nutritional value of eggs for human consumption.

## Findings

### Background

Eukaryotic multicellular life has evolved a variety of techniques in order to nourish the developing young both before and after birth, these techniques include nourishment via the placenta, the avian and reptilian egg and mammalian lactation. Whilst the primary function of these various adaptations is nutrition they also aid development through the delivery of bioactive molecules such as hormones [1, 2], antibodies [3, 4] and, in the case of mammalian milk, through the delivery of microRNAs (miRNA) [5].

These miRNAs are a small non-coding species of RNA found in eukaryotic cells that play an important role in gene regulation and are involved in a diverse variety of biological processes [6]. These regulatory molecules are found in a number of biological fluids including blood, milk and saliva [7, 5, 8]. There is evidence that the poultry egg packages bioactive molecules, such as hormones and antibodies, which play an important role in the epigenetic development of the chick [9–11].

Despite a number of studies having profiled miRNA expression in the developing chicken embryo [12], to our knowledge, no-one has yet described the presence of miRNA in either the albumen (white) or yolk of the chicken egg. In this study we describe a method for reliably and reproducibly extracting miRNA from both the albumen and yolk of unembryonated chicken eggs. We go on to screen the extracted RNA for the presence of 21 miRNAs via reverse transcription quantitative real-time PCR (RT-qPCR). Of these 21 miRNAs, ten were selected as they had previously been described to be present in the developing chick [12–14], a further ten were selected as they have been found to be expressed in chicken tissue from unrelated work in our lab (unpublished) and a housekeeping putative miRNA. Following detection of miRNA in both albumen and yolk we analyse the expression pattern of five of these microRNAs in greater detail and show differences between the yolk and albumen in respect to the levels of specific miRNAs.

## Methods

### Extraction of miRNA from albumen

Reagents, unless otherwise indicated, were those supplied with the Exiqon© miRCURY™ biofluid RNA isolation kit for both the albumen and yolk extractions. Initial extractions were checked for purity for both the albumen and yolk extractions by analysing the absorbance ration A260/A280 and were all found to be >1.9. Albumen from two commercial unembryonated cage-laid eggs were homogenised by vortexing and 200 µl of homogenate was then diluted in lysis solution BF at a 1:1 ratio, vortexed thoroughly and incubated at room temperature for 15 min. Fifty units of proteinase K (Promega), at a concentration of 10,000 units per ml in sterile distilled H_2_O, was added and the solution vortexed and incubated at 37 °C for 10 min. The solution was centrifuged at 3000*g* for 5 min. The clear supernatants was transferred to a new 1.5 ml microcentrifuge tube and to this 40 µl of protein precipitation solution BF was added and mixed by vortexing followed by incubation at room temperature for 1 min. The solution was then centrifuged for 3 min at 11,000*g*. After this the extraction continues as per the Exiqon© miRCURY™ biofluid RNA isolation kit standard instructions from step 4 onwards.

### Extraction of miRNA from yolk

Yolk from two commercial unembryonated cage-laid eggs were homogenised by vortexing and 1 ml of homogenate was then diluted in lysis solution BF at a 1:1 ratio, vortexed thoroughly and incubated at room temperature for 15 min. 400 µl aliquots of the yolk/lysis solution was dispensed into five 1.5 ml microcentrifuge tubes. To each of these aliquots 600 µl of Cleanascite™ was added followed by rigorous vortexing until the sample became homogenous. The Cleanascite™ removes the lipid from this high fat tissue that would otherwise interfere with the extraction process. The ratio of Cleanscite™:yolk is 3:1 (taking into account that the yolk has been diluted in an equal volume of lysis solution BF). During optimisation of this procedure ratios of Cleanascite™:yolk between 1:1 and 1:10 were performed with 1:3 being found to give the best balance between yield and purity. Solutions were then incubated at 4 °C for 1 h. After incubation samples were centrifuged at 13,000*g* for 5 min. Clear supernatant was transferred to a new 1.5 ml microcentrifuge tube and to this 40 µl of protein precipitation solution BF was added. The solution was mixed by vortexing followed by incubation at room temperature for 1 min and then centrifugation for 3 min at 11,000*g*. After this the extraction continues as per the Exiqon© miRCURY™ biofluid RNA isolation kit standard instructions from step 4 onwards.

### RT-qPCR of miRNA from extracted RNA

The miRNA was quantified using the Qubit^®^ MicroRNA Assay Kit on a Qubit^®^ 3.0 Fluorometer using software APP v1.02 + MCU v0.21. Primers and probes were purchased from Applied Biosystems™ from their TaqMan^®^ MicroRNA assay range, manufactures probe name and the name of the equivalent published chicken miRNA, when applicable, (as per mirBase Release 21) can be found in Table 1 along with the sequence of the housekeeping gga-mir-control.

**Table 1:** TaqMan^®^ probes used in this study

miRNA (probe name)	miRNA (chicken annotation)	Presence	
		Yolk	Albumen
hsa-mir-10a	gga-mir-10a-5p	**–**	**–**
hsa-mir-10b	gga-mir-10b-5p	**–**	**–**
hsa-mir-22	gga-mir-22-3p	**–**	**–**
hsa-mir-26a	gga-mir-26a-5p	**–**	**–**
hsa-mir-30c	gga-mir-30c-5p	**+**	**+**
hsa-mir-30c-1	No published equivalent	**–**	**–**
gga-mir-30c-1-3p	n/a	**–**	**–**
hsa-mir-92a	gga-mir-92-3p	**+**	**+**
hsa-mir-99a	gga-mir-99a-5p	**+**	**–**
hsa-mir-100	gga-mir-100-5p	**+**	**–**
mmu-mir-142-3p	gga-mir-142-3p	**+**	**–**
gga-mir-146c	n/a	**–**	**–**
hsa-mir-183	gga-mir-183	**–**	**–**
hsa-mir-204	gga-mir-204	**–**	**–**
hsa-mir-215	gga-mir-215-5p	**+**	**+**
hsa-mir-486-3p	No published equivalent	**–**	**–**
oan-mir-1388-5p	No published equivalent	**–**	**–**
gga-mir-2188	n/a	**+**	**+**
tgu-mir-2970-5p	No published equivalent	**–**	
hsa-let-7f	gga-let-7f-5p	**–**	
gga-mir-control	5′–CCGAGGCGCCUCGGUGGGC–3′	**+**	**+**

**Fig. 1:**
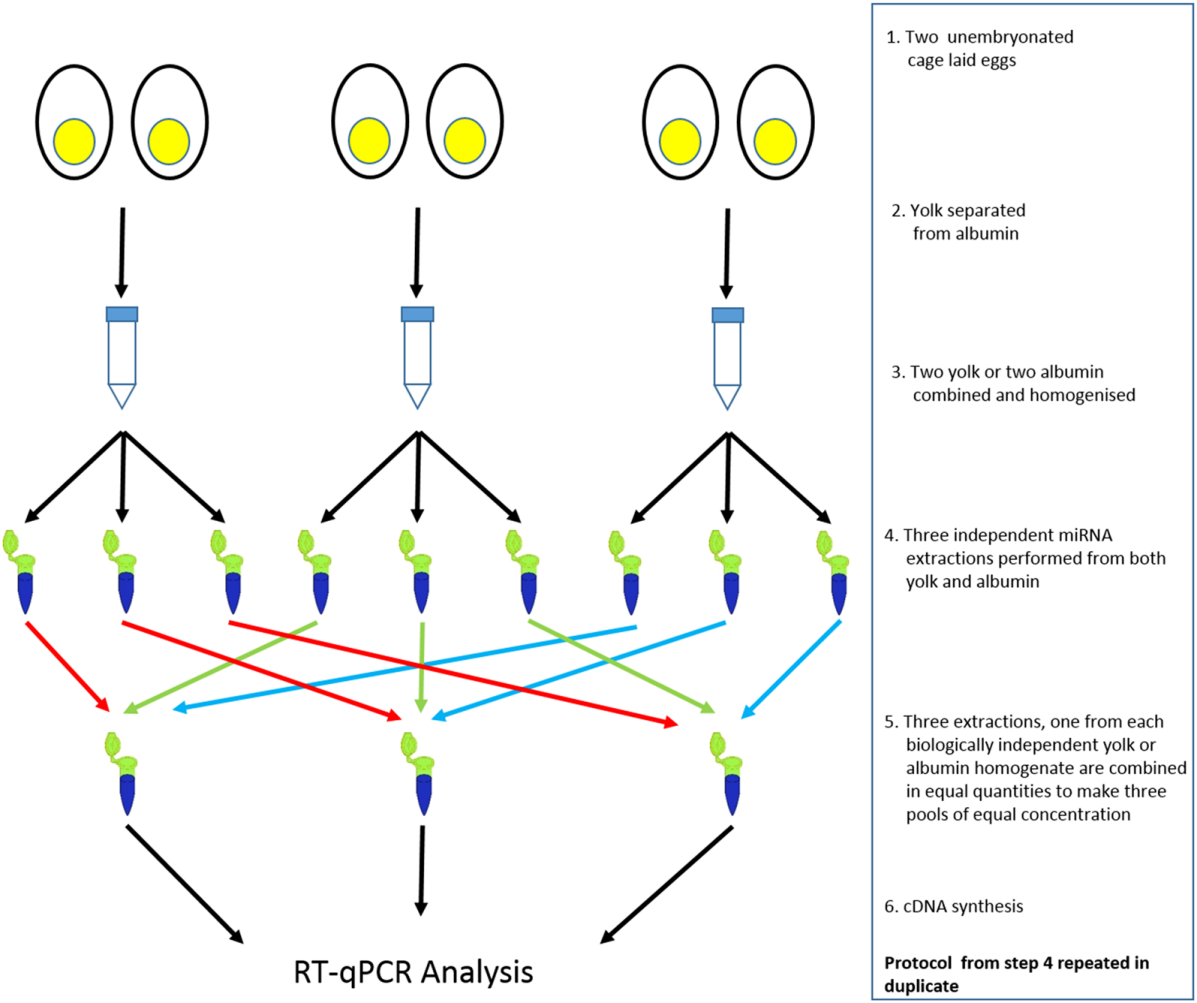
Protocol for the generation of pooled miRNA. A *flow*-*diagram* detailing the generation of the pooled miRNA used for cDNA synthesis. A total of nine extractions from three biologically distinct homogenates (comprising of either the albumen or yolk of two eggs) is used to produce the three pools. The protocol is repeated in duplicate

The method of producing the miRNA pools subsequently used in the RT-qPCR analysis is shown in Fig. 1. The rationale behind this method was firstly to show that the method worked consistently in extracting miRNA (total of 18 extractions from either fluid) and secondly, by pooling the extractions, to demonstrate reproducibility by minimising the potential impact of any single egg with a radically different miRNA profile from the others. Briefly the yolk and albumen of two eggs are separated and the two yolks and two albumen homogenised together, this is done three times to produce three biologically distinct homogenates. From each homogenate three independent extractions were performed and one extraction from each homogenate are combined in equal quantities to generate the three pools. This protocol was performed in duplicate.

10 ng of pooled miRNA was used in a 15 µl complimentary DNA (cDNA) synthesis reaction using the Taqman^®^ microRNA reverse transcription kit (Applied Biosystems™) as per manufacturer’s instructions in conjunction with the appropriate primers, no more than six miRNAs were amplified in a given cDNA reaction. The RT-qPCR reactions were formulated as per manufacturer’s instructions using 1 µl of the above cDNA per 15 µl reaction and using Taqman^®^ universal master mix II, no UNG (Applied Biosystems™). RT-qPCR was undertaken on a Bio-Rad C1000 thermal cycler with CFX96 real-time system with the following protocol; 10 min at 95 °C followed by 40 cycles of 95 °C for 15 s and 60 °C for 1 min with fluorescence being read at the end of the 60 °C step. Acquisition and analysis of data was undertaken using the software BioRad CFX manager version 3.0.1215.0601. Ct values were recorded at a relative fluorescent unit value of 20,000. Expression levels were calculated relative to gga-mir-control where the average expression of gga-mir-control across the yolk pools was given an arbitrary expression value of 1000. Each of the 6 gga-mir-control values for both tissue were then scaled against the average across the yolk pools allowing normalisation against the control within each sample.

Statistical analysis was undertaken using SPSS version 23. Statistical significance was calculated using One-Way ANOVA with Tukey Post Hoc test.

## Results and discussions

### MiRNA can be extracted and detected from albumen and yolk

This is the first report of the extraction of miRNA from the albumen and yolk of chicken eggs. One reason why this may not have been attempted before could be the difficulties presented by the composition of these two tissues; with yolk being lipid rich and albumen high in protein. We overcame these potential problems by proteinase K treatment of the albumen and Cleanascite™ treatment, which binds and removes lipids from samples, of the yolk.

Eighteen independent extractions were undertaken from the albumen and yolk of commercial cage-laid eggs. The extractions were quantified using a miRNA specific quantification assay and water only extractions were included as negative controls. Albumen extractions ranged in concentration from 72 to 2.21 ng/µl in a total volume of 50 µl making for a total yield of between 3.6 and 110.5 ng of miRNA from 200 µl of starting albumen with an average concentration of 595 pg/µl (average total yield of 29.75 ng). The concentration of the yolk extractions was between 243 pg/µl and 5.37 ng/µl in a total of 50 µl making for a yield of between 12.15 and 268.5 ng of miRNA from 1 ml of starting yolk giving an average concentration of 2.33 ng/µl (average total yield of 116.5 ng). In all cases the water only negative control did not register as containing miRNA in the quantification assay.

To confirm that these concentrations translated to detectable miRNA and to demonstrate that such extractions were suitable for downstream application we chose to perform RT-qPCR using cDNA generated from the six pools (see Fig. 1) of miRNA for each fluid against a suite of 21 target miRNAs. Initially all 21 targets were investigated in just a single albumen and yolk pool before performing a more targeted investigation. A miRNA was considered ‘present’ if it was detected prior to the 35th cycle in the RT-qPCR run. Although signal could be detected for some probes beyond cycle 35 the signal tended to be inconsistent with large differences between technical duplicates and were considered generally unreliable so an exclusive rather than inclusive approach was taken. Results are shown in Table 1. Of the 21 miRNAs screened for eight were present in yolk and five in albumen. The five present in albumen were all present in yolk.

**Fig. 2:**
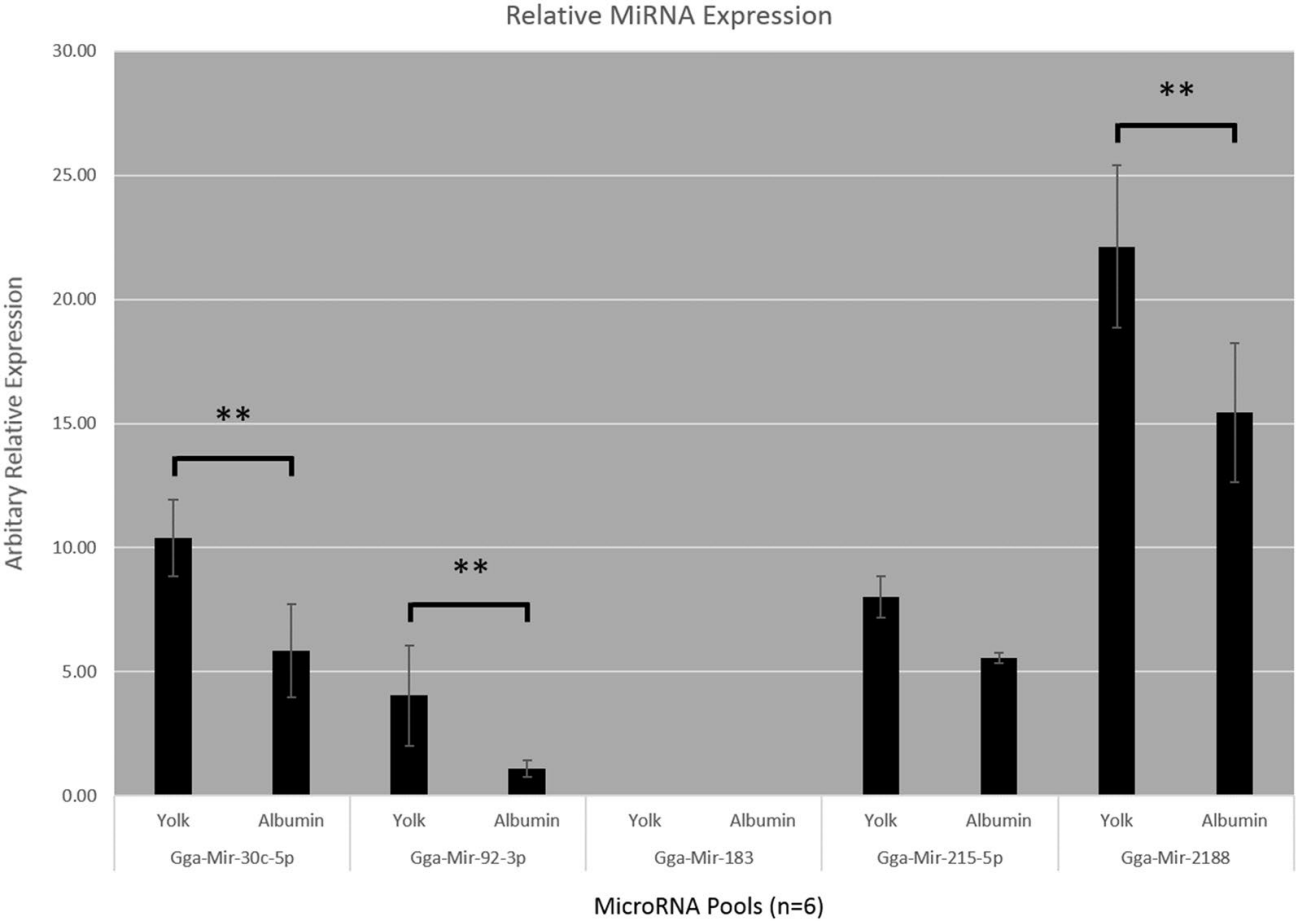
Relative expression levels of five miRNAs in yolk and albumen. Average expression of miRNAs across six pools for yolk and albumen in arbitrary units relative to gga-mir-control. *Error bars* represent standard deviation. *Black bars* represent statistically significant differences between the level of average relative expression of that particular miRNA between yolk and albumen pools as per one-way ANOVA with Tukey post Hoc test. **denotes *p* < 0.01

Four targets and two controls were chosen for expression analysis across all pools. These were gga-mir-control, as an endogenous positive control and for calculating relative expression; gga-mir-183, which was not present in either fluid, as an endogenous negative control; gga-mir-2188, gga-mir-30c-5p, gga-mir-215-5p and gga-mir-92-3p all of which were present in both fluids. Results of the analysis are shown in Fig. 2. All miRNAs previously detected in both fluids were found to be consistently present and gga-mir-183 was not detected in any sample. The relative expression of gga-mir-2188, gga-mir-30c-5p and gga-mir-92-3p was significantly (*p* < 0.01) higher in yolk than in albumen.

This is the first study to report the isolation and detection of miRNAs in either chicken egg albumen or yolk. This observation means that chicken egg albumen and yolk join mammalian milk [5] as being a nutritive substance which also carries miRNAs. The methods of extraction reported here will be of value to both veterinary and miRNA researchers particularly in respect to those with an interest in the epigenetic factors that influence livestock production.

It is interesting to note that seven of the eight miRNAs detected within the egg, all have either been previously reported to be expressed in embryos or have been directly implicated in embryo development. The equivalent namesake mouse and zebrafish miRNAs of gga-mir-2188, gga-mir-99a-5p and gga-mir-142-3p have been shown to be involved in the development of the vascular system and hematopoiesis in the embryos of mice and zebrafish [15–17]. The mir-30 family of miRNAs have been shown to be involved in early muscle development in zebrafish [18] and the mouse equivalent (mmu-mir-100-5p) of gga-mir-100-5p is involved in embryonic stem-cell differentiation [19]. Whilst in humans hsa-mir-99a and hsa-mir-92a are necessary for correct embryo development [20, 21]. Finally in pigs ssc-mir-215-5p has been reported to be present in the head and organs of 33 day old embryos. Of the seven miRNAs three; gga-mir-30c-5p, gga-mir-99a-5p and gga-mir-100-5p have been reported to be present within chicken embryos [14].

It is possible that the presence of miRNA in the egg is simply a by-product of egg development/maintenance and reflects the processes involved therein and are not actually playing a role in chick development *per se*. Arguing against this though is the fact that albumen and yolk of unembryonated eggs display very low levels of metabolic activity [22, 23] and as such would be unlikely to be producing large amounts of miRNA. Given our observation of the presence of seven miRNAs within the egg that have either been implicated in embryo development or shown to be expressed in embryos of chickens and other species it would seem more likely that at least some of the miRNAs in these fluids are playing a role in embryo development rather than being reflective of more general egg processes. Whilst we acknowledge that we can only speculate at this point, our miRNA extraction method will enable further research to investigate such hypotheses.

The discovery of miRNAs in eggs has potentially important implications. The active transport of beneficial miRNAs from the mother into the albumen or yolk would be an efficient mechanism for the chicken to help promote normal chick development as the miRNAs could be continually supplied to the growing embryo during nutrient uptake. If, as we suggest, maternally derived miRNAs in the egg are being used during chick development then this presents an attractive manner in which the poultry industry may be able to promote better production standards and animal welfare outcomes. The selective inclusions of miRNAs into the egg that promote immune system development or increased intestinal absorptive efficiency are just two examples of the possible applications of this exciting discovery. Another potential outcome of these findings could be the improvement of eggs for human consumption by modulating nutritional value or extending their shelf-life.

## Abbreviations

miRNA: microRNA
RT-qPCR: real-time PCR
cDNA: complimentary DNA
